# Spatiotemporal Analysis of Infant Measles Using Population Attributable Risk in Shandong Province, 1999–2008

**DOI:** 10.1371/journal.pone.0079334

**Published:** 2013-11-19

**Authors:** Yuhui Zhu, Qing Xu, Hualiang Lin, Dahai Yue, Lizhi Song, Changyin Wang, Huaiyu Tian, Xiaoxu Wu, Aiqiang Xu, Xiujun Li

**Affiliations:** 1 Department of Epidemiology and Health Statistics, School of Public Health, Shandong University, Jinan, China; 2 Shandong Provincial Center for Disease Control and Prevention, Jinan, China; 3 Guangdong Provincial Institute of Public Health, Guangdong Provincial Center for Disease Control and Prevention, Guangzhou, China; 4 China Center for Health Development Studies, Peking University, Beijing, P. R. China; 5 School of Environment, Tsinghua University, Beijing, P. R. China; 6 College of Global Change and Earth System Science, Beijing Normal University, Beijing, P. R. China; Centers for Disease Control and Prevention, United States of America

## Abstract

**Background:**

Reduction of measles incidence and mortality has been encouraging in China. However, it remains an important public health concern among infants. This study aimed to examine the space–time distribution pattern of infant measles occurrence for the period of 1999–2008 in Shandong, China.

**Methods and Findings:**

Measles cases among infants aged younger than 1 year were obtained from the national infectious diseases reporting information system. A spatiotemporal analysis using population attributable risk percent (PAR%) was used to distinguish between multiple geographic clusters of potential interest. The analysis detected 29 statistically significant space–time clusters with the most likely cluster in Zaozhuang City from 2006 to 2008. Of the 28 secondary clusters, 22 were found in 2008. The map of PAR%, relative risk (RR) and space–time cluster analysis indicated that the clusters were generally unchanged, and were found south-west and north-west of Shandong. The Lanshan District in Linyi had the highest PAR%, while highest RR was in the Yicheng District in Zaozhuang.

**Conclusion:**

There were significant space-time clusters of infant measles in Shandong over the study period. PAR% is an effective way to analyze multiple clusters from their application like RR. Interrupting measles circulation and maintaining routine coverage over 95% may be the only effective strategy to achieve measles elimination.

## Introduction

Measles is one of the most important contagious vaccine-preventable diseases and causes millions of pediatric deaths worldwide. As the goal of World Health Organization, reduction of measles incidence and mortality has achieved significant progress due to widespread use of measles vaccine since the 1980s [1]. The measles vaccine coverage among children by their first birthday has increased to 84% in 2011 from 72% in 2000, which was mainly attributed to the improved routine health service [2]. As a result, the measles mortality has decreased from 548,300 deaths in 2000 to 157,700 in 2011. However, re-emergence of measles cases has been of increasing concern in recent years. Large scale outbreaks have been reported in vaccinated population in recent years [3]–[5]. Moreover, an increasing proportion of infants have been affected [6]–[7].

In China, the incidence of measles decreased remarkably after the introduction of liquid measles vaccine (MV) in 1965. Since 1986, a 2-dose schedule using measles-containing vaccine, which is the first measles vaccine included in the National Expanded Program on Immunization (EPI), has been administered to infants aged >8 months for the first dose and aged 7 years for the second dose according to the recommendation of the Center for Disease Control and Prevention (CDC) [8]. The overall number of reported cases decreased from 73,567 in 2000 to 52,461 in 2009 in China, while the number of infant cases increased from 5,184 in 2000 to 16,969 in 2009 [9]. Consequently, infant measles has become a key obstacle to the achievement of the target of 95% reduction of measles mortality by 2015, compared with 2000.

Previous studies have suggested that the risk of measles varies geographically. The disease is distributed worldwide and there are large differences between regions. The analysis of spatial and temporal variations in the incidence of measles can be useful for optimizing measles surveillance and control efforts in areas where they are most needed [10]. The spatial scan has been increasingly used as a useful tool to explore the spatial and temporal clusters and to formulate new etiological hypothesis [11]–[13]. However, it has difficulty in distinguishing statistical and public health significant regions when multiple clusters are detected. One potential problem is that these clusters are ranked by their likelihood-ratio-based test statistics. The ranking may neglect the region where relative risk is only moderately high, but population size is large. In epidemiological study, attributable risk is a more relevant indicator for exposure-disease association than the excess risk, relative risk, or odds ratio [14]. Its variants can provide us the contribution of a risk factor to overall incidence or prevalence of disease. Previous analyses of measles dynamics have shown birth rates can affect multi-annual outbreak dynamics [15]. Therefore, using population attributable risk percent (PAR%) can further interpret the clusters based on local population. And the PAR% can also be useful in public health intervention for the total burden of disease [16].

In this study, we collected the information of infant measles in Shandong Province during 1999–2008 using PAR% for space-time cluster analysis. We aimed to examine the space–time cluster pattern of infant measles, which would help to identify areas and population at high risk and also to draft suitable prevention and control measures in this province.

## Materials and Methods

### Ethics Statement

This study was approved by the Human Research Ethics Committee, Shandong University.

### Setting

**Figure 1:**
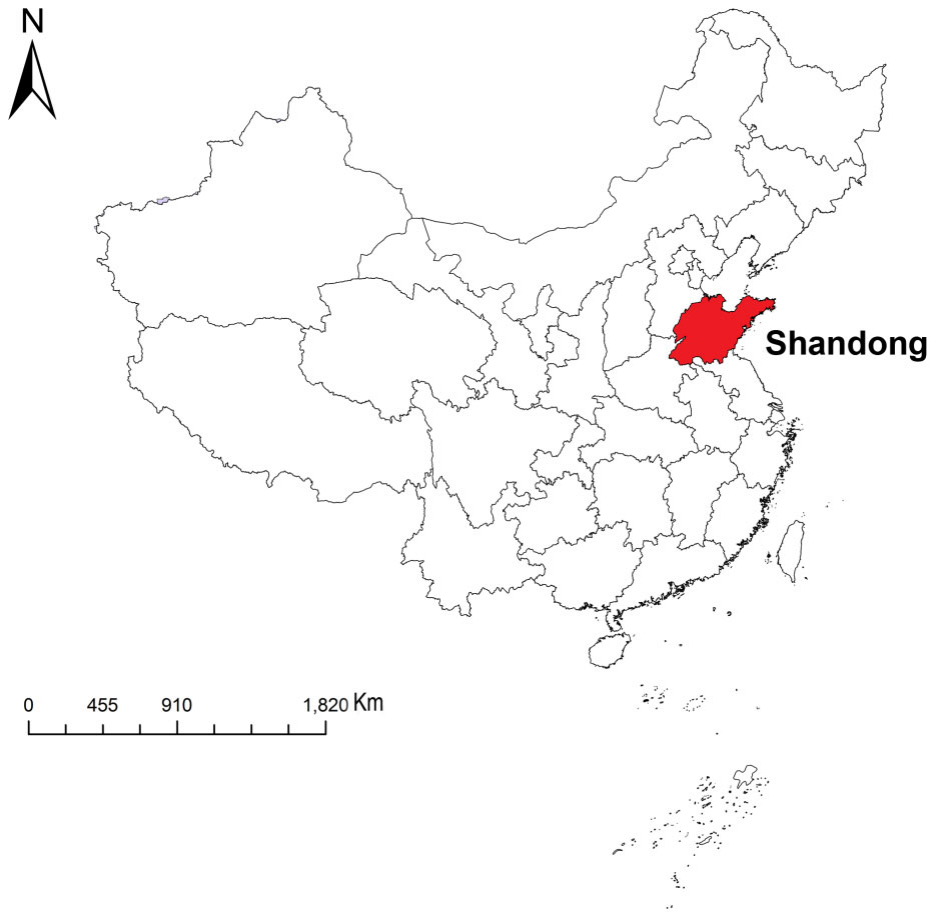
Location of Shandong Province in China.

Shandong Province is located in the lower reaches of the Yellow River, on China's eastern coast line, with a total area of 157,100 km^2^ and a total population of approximately 96 million in 2010. Shandong is divided into 17 administrative divisions, which are subdivided into 142 counties/districts. The province consists of two distinct segments - the inland zone and the Shandong Peninsula. The inland zone includes a hilly central region and a fertile agricultural area on the north, west and south. On the contrary, the Shandong Peninsula is entirely an upland area with fishing, mining and port-related activities as the major economic sources. Shandong has a continental climate characterized by cold winters and hot, dry summers. Climate variation is much different between the peninsular and inland zones of the province (Figure 1).

### Data Sources

Measles is a notifiable infectious disease in China; any measles case must be reported to the local CDC according to the National Regulation of Infectious Diseases. A case definition is any patient with rash and fever and having at least one of the three symptoms: cough, coryza and conjunctivitis [17]. We collected measles cases of infants aged <1 year old and obtained from the national infectious diseases reporting information system of the Shandong CDC for the period 1999–2008. The data included information of age, gender, residential address, and reporting month. Relevant demographic data and electronic boundaries of each district were retrieved from the local Statistics Bureau. District information included district name, code, digital boundaries and a base map (1:1,000,000 scale).

### Space–Time Analysis

We used SaTScan software (version 9.1.1) to search for measles clusters in infants by space-time statistical analyses. Since the data were based on an aggregation of cases and population into census divisions, we fitted the Poisson model likelihood ratio test for the cluster detection [18]. For cylindrical scanning window, we set a maximum spatial cluster size of 50% of the population at risk and a maximum circle radius of 50-km in the spatial window, and a maximum of 50% of the study period in the temporal window. Considering pre-selection bias as described in the SaTScan User Guide, the results obtained to the 50% of the population limit were very similar to 10% of total population at risk [19]. Fifty km was chosen as the maximum circle radius for all analyses because of all districts were within a 50-km radius. Space-time analyses were also conducted with 30-km, and the results obtained were very similar to 50-km results. In this study, we allowed primary and secondary clusters to overlap. The primary cluster was most-likely to cause the rejection of a null hypothesis of constant risk in space and time. Significant clusters were evaluated with P<0.05 based on Monte Carlo 9999 replications.

### Attributable Risk Clusters

Attributable risk considers both the magnitude of association between exposure and outcome and the size of the population exposed, which is unlike relative risk that doesn't take into account the actual number of cases of disease which might be related to a given exposure to risk [20]. In cluster detection applications, clusters with high relative risk may represent the probability of being a case and probability of being inside a cluster is high. However, clusters with highest test statistics may or may not be associated with higher values of relative risk [16]. Attribute risk is used to identify which clusters, among a number of statistically significant clusters, contains the largest number of cases resulting from being inside the cluster. Therefore, when multiple clusters were found by SaTscan, and these clusters differed with respect to relative and attribute risk, it was essential to understand variation in disease burden between places.

Population attributable risk percent (PAR%), which depends partly on the prevalence of risk factors in the population, is defined as the proportion of disease in the population that is due to a given risk factor. The PAR% is estimated by [21]

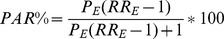
where P_E_ is the proportion of the total number of exposed population that is used the native birth data. And RR is the relative risk of disease associated with exposure that relies on the results of SaTscan software.

## Results

### Cases and Incidence Rates

**Figure 2:**
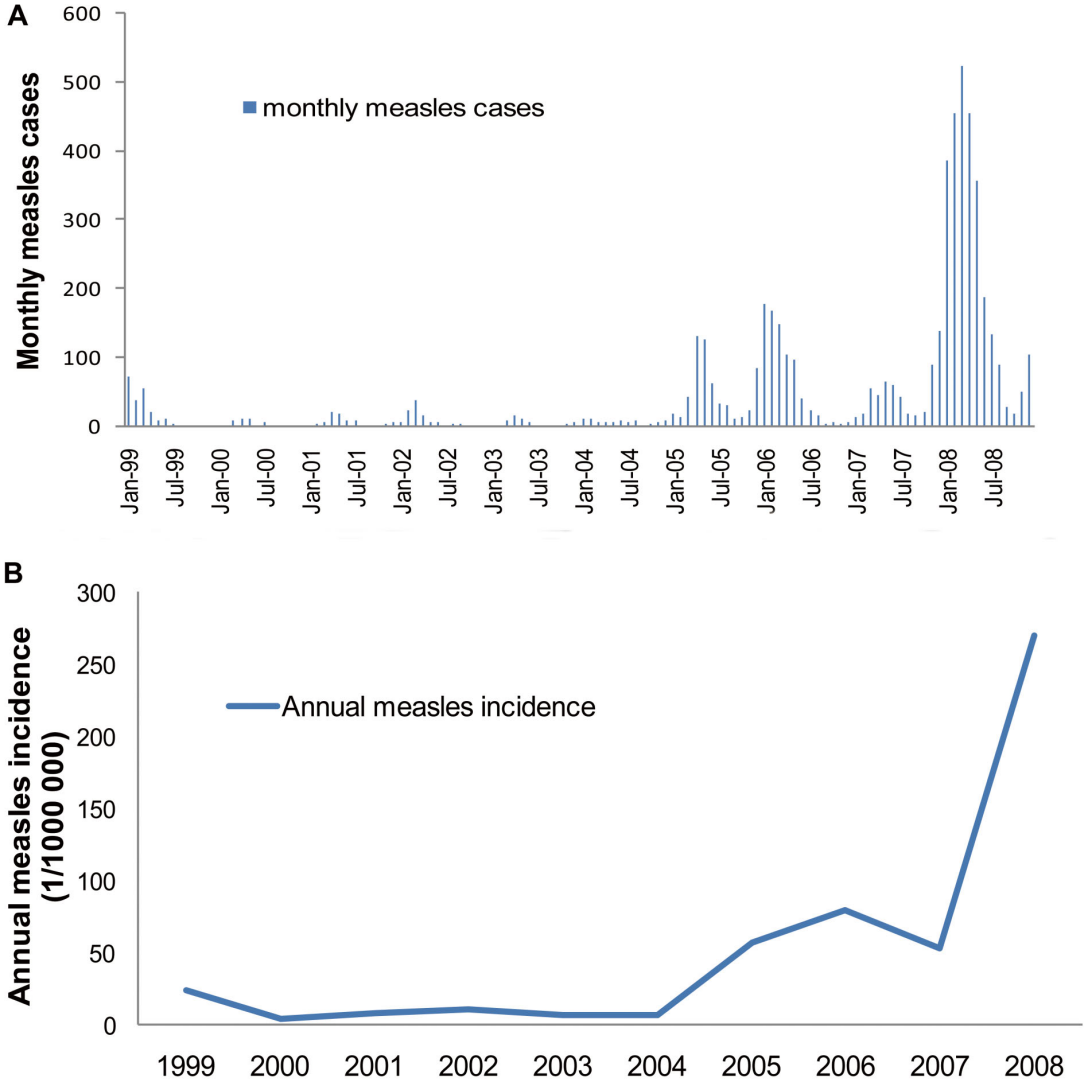
The epidemic pattern of measles in infants, Shandong, China, 1999–2008. A) Monthly cases and the seasonal epidemic patterns. Average monthly epidemic curves indicate the epidemic peaks of measles. B) Annual incidence rates show two notable shifts of measles yearly incidence.

Figure 2 showed variation in monthly numbers of districts with measles infection from 1999 to 2008. A total of 5,309 cases were reported in infants less than 1 year of age. Most (2,791/5,309) cases were reported in 2008, and an additional epidemic peak was found in 2006 (792/5,309). The highest incidence rate of 269.6 per 100,000 was observed in 2008 and the lowest incidence rate of 4.16 per 100,000 was found in 2000. Of the reported cases, 68% were male and 32% were female. The monthly epidemic peaks appeared during March to May.

### Spatio-Temporal Clusters

**Figure 3:**
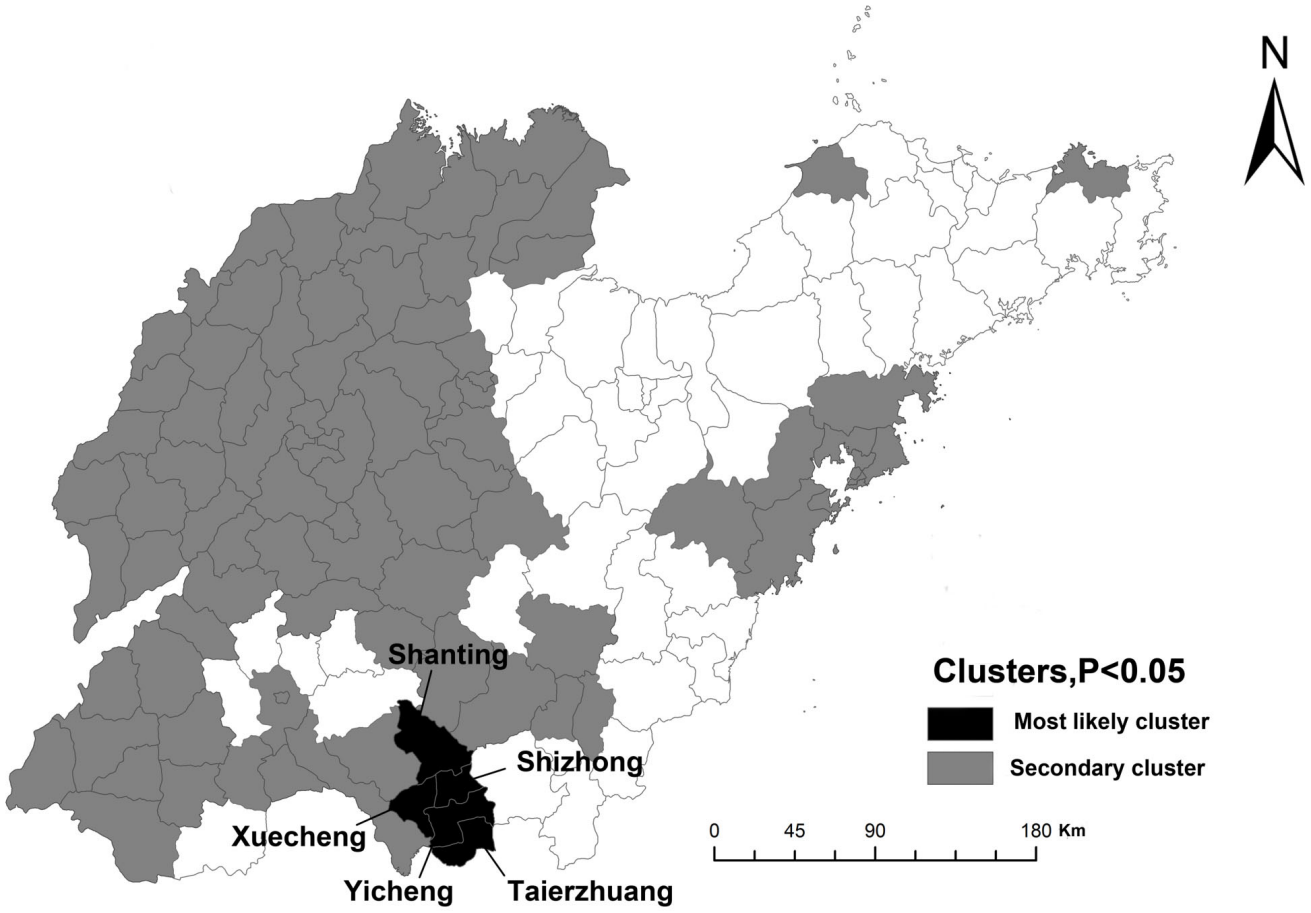
Space-time significant clusters of infant measles identified in Shandong, 1999–2008. The most likely cluster in black is located in Zaozhuang City. Grey parts display the secondary clusters and counties in white on the map have no clusters.

The space-time cluster analysis detected a total of 29 statistically significant space–time clusters (Table 1, Figure 3). Table 1 showed the number of county/district included in each cluster, time frame, radius (km), observed cases, expected cases, relative risk (RR) and log-likelihood ratio. The most likely cluster included 5 districts (Shizhong, Xuecheng, Yicheng, Shanting, Taierzhuang) sited in Zaozhuang City, the south of Shandong Province (Figure 3). In this area, 599 observed cases were compared to 43.42 expected cases from 1 January 2006 to 31 December 2008 (RR = 15.42, Radius = 37.82). Of these secondary clusters, year 2008 had 22 clusters (RR ranged from 2.35 to 37.76) and the highest risk was detected for Hedong and Lanshan Districts in Linyi City. Secondary clusters were concentrated mostly in the west (Figure 3).

**Table 1:** Retrospective space-time clusters of infant measles in Shandong, 1999–2008.

Cluster	No. districts	Time frame	Radius (km)	Observed	Excepted	RR	LLR*
1†	5	2006/1/1 to 2008/12/31	37.82	599	43.42	15.42	1046.77
2	2	2008/1/1 to 2008/12/31	15.12	341	9.61	37.76	895.46
3	11	2008/1/1 to 2008/12/31	45.21	475	36.82	14.07	795.29
4	8	2007/1/1 to 2008/12/31	42.47	479	50.74	10.28	665.04
5	8	2008/1/1 to 2008/12/31	49.07	288	26.23	11.55	434.89
6	2	2008/1/1 to 2008/12/31	4.18	177	6.82	26.82	408.95
7	6	2008/1/1 to 2008/12/31	43.01	250	30.53	8.54	310.83
8	1	2005/1/1 to 2006/12/31	0	149	9.03	16.95	279.64
9	8	2006/1/1 to 2008/12/31	42.38	323	79.40	4.27	215.39
10	6	2008/1/1 to 2008/12/31	32.26	150	14.65	10.51	215.32
11	4	2008/1/1 to 2008/12/31	36.44	150	15.06	10.22	211.56
12	3	2008/1/1 to 2008/12/31	33.83	168	21.21	8.15	202.96
13	6	2008/1/1 to 2008/12/31	46.79	119	8.92	13.63	199.43
14	5	2006/1/1 to 2008/12/31	49.06	283	72.36	4.07	179.60
15	7	2008/1/1 to 2008/12/31	45.11	138	20.64	6.84	146.13
16	7	2008/1/1 to 2008/12/31	25.50	110	14.11	7.94	130.86
17	5	2008/1/1 to 2008/12/31	41.86	160	39.12	4.19	105.89
18	2	2008/1/1 to 2008/12/31	37.90	97	16.38	6.01	92.52
19	4	2008/1/1 to 2008/12/31	41.84	80	12.50	6.48	81.43
20	4	2005/1/1 to 2008/12/31	49.42	168	56.95	3.01	71.88
21	3	2008/1/1 to 2008/12/31	32.09	101	24.21	4.23	68.02
22	4	2008/1/1 to 2008/12/31	42.41	77	22.56	3.45	40.36
23	2	2008/1/1 to 2008/12/31	27.12	39	12.23	3.20	18.52
24	1	2008/1/1 to 2008/12/31	0	30	8.55	3.52	16.26
25	5	2008/1/1 to 2008/12/31	47.49	49	20.95	2.35	13.66
26	1	2008/1/1 to 2008/12/31	0	14	2.41	5.82	13.06
27	5	2008/1/1 to 2008/12/31	49.91	44	18.72	2.36	12.39
28	4	2008/1/1 to 2008/12/31	31.91	41	17.02	2.42	12.12
29	1	2005/1/1 to 2006/12/31	0	14	2.61	5.37	12.12

RR, relative risk; LLR, log-likelihood ratio.

* P<0.05.

† Most likely cluster.

### Attributable Risk Clusters

**Figure 4:**
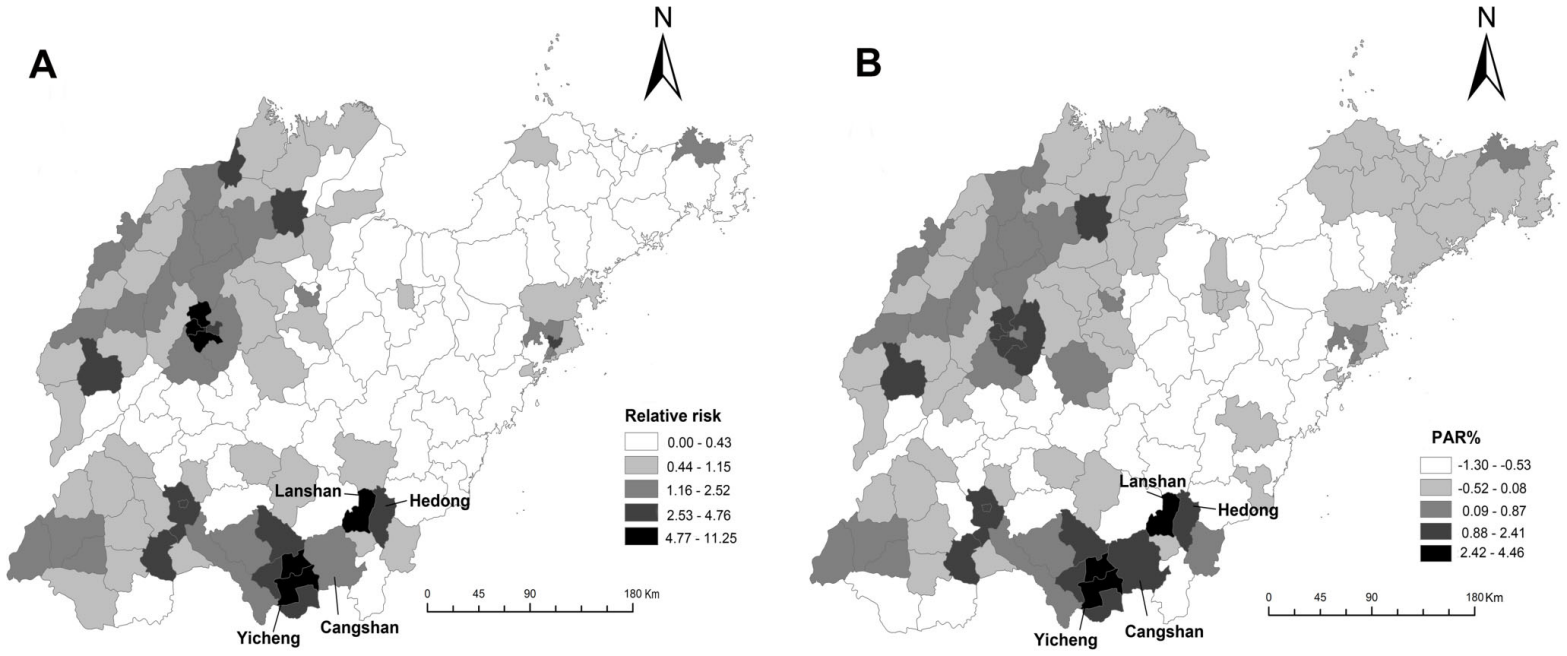
Methods of measles cluster detection in endemic areas of Shandong Province, 1999–2008. A) Relative risk (RR) of infant measles. B) Population attributable risk percent (PAR%) of infant measles.

Figure 4 showed the map of infant measles RR and PAR% in Shandong Province during 1999–2008.The Yicheng District in Zaozhuang City had the highest relative risk at 11.25 and considerably high PAR% at 4.43, which meant that 4.43% of the infant cases in Yicheng could have been prevented if it had the same social, environmental and other district-related risk factors as the rest of the regions. On the other hand, the Lanshan District of Linyi City had the highest PAR% at 4.46 and RR estimate was also far beyond the normal at 5.61. For those with low PAR%, the negative results meant the possibility of the absence of risk factors or presence of protective factors. Compared to the Figure 3, the clusters were mostly unchanged, and were found in south-west and north-west. Although the results obtained from these analyses were generally similar, some differences should be observed carefully. We found Cangshan County in Linyi City had a high PAR% at 1.12, but with RR only slightly greater than 1.00 (1.55).

## Discussion

To our knowledge, this is the first study to analyze the space–time risk of infant measles using PAR%, which could enhance our understanding of spatial clusters. The results from PAR%, RR and space–time cluster analysis suggested a similar space–time pattern in the distribution of infant measles, which was clustered in the southwest and northwest parts of Shandong. For example, Zaozhuang city included in the most likely cluster and one of its counties, Yicheng, had significantly high PAR% and RR. The fact that risk was located around the same areas may suggest the possible relationship between the geographic locations of environmental or social factors, such as precipitation, temperature change, humidity, socio-economic status of local residents, population movements, vaccination coverage and different surveillance networks in these regions [22]. Zaozhuang is located in the south of Shandong Province, near Jiangsu Province. Its vaccination coverage was relatively low. Of the 1626 measles cases during 2004–2008, more than half (57.01%) were not immunized [23].

At the same time, compared with RR, PAR% has its own advantages. The Lanshan District in Linyi had the highest PAR%, while highest relative risk was in the Yicheng District in Zaozhuang. When analyzed in terms of RR in Cangshan, the result of measles clusters was not particularly significant. However, based on PAR%, there was an obvious pattern which indicated an increasing geographic concentration of risk. Cangshan County had a high population density with 195,288 infants born during 1999–2008. So the advantage of using PAR% is that it can supplement the outcome of clusters regarding the clusters with high birth rate. Given that the ability to estimate the reduction in disease that may occur if particular risk factors are eliminated, PAR% may be an effective method for place-specific intervention in disease burden [24].

Most of the significant clusters included year 2008. Weakening the herd immunity by gradual accumulation of measles susceptible individuals in the population could be the reason for the increased incidence of infant measles. This is in agreement with a review that the decay of measles antibodies can be a result of the increasing age of first childbirth and fewer exposures to natural viruses received by the young adults [25]. Several studies indicated that infants too young to get immunized were at increased risk because of losing maternal antibodies before the initiation of the immunization schedule [26]–[28]. That is, immunity of infants is less robust and less durable provided by vaccine-induced mothers. Since the effort of MV and EPI, most women of childbearing age in China did not contract vaccine-preventable diseases in childhood, but were immunized instead. From the data of Shandong population monitoring results during 1999–2004, the antibody-positive rate of measles was 90.71% and the geometric mean titre (GMT) of measles IgG antibody was 1:569.54 [29]. A survey also found that the level of measles IgG was lower in 50.37% of pregnant women [30]. In addition, on the basis of measles dynamics for industrialized countries, high birth rate regions should experience regular annual epidemics [31]. It is in accord with outbreaks of measles every 3–4 years in China [9]. And history data have shown that the rising morbidity of the whole crowd measles is followed by the rising morbidity of infant measles. Remarkably, a case-control study of 84 infants reported that 49 infants had hospital exposure history and it was an important risk factor for infant infection (OR = 15.4), which suggested that control of hospital infection cannot be ignored [32].

However, there are no measles clusters during 1999–2004. Several reasons might help to explain this result. One possible reason is that the national network of direct reporting system was established in 2004 and completed in 2005. The disease information during 1999–2004 might have been underestimated due to the possible incomplete reporting. Another reason may be the measures taken in 1999 and 2001, respectively. It required 8 months to 7-year-old and 7–14 year-old to carry out measles immunization, which enhanced the population immunity [33]. This could indicate that population-based public health interventions might be warranted.

A few limitations of this study need to be considered. First, the exact onset date of symptoms was not available, only the year and month of the notification was recorded. Meanwhile, our study was not able to include the seroprevalence for each region. However, according to the seroprevalence survey of 1999–2005, H1 genotype was the predominant virus in Shandong Province [34]. Second, we only identified potential cluster areas according to the exploratory study, but did not link possible risk factors accounting for these clusters. Further investigations are needed to discover the underlying mechanisms of increased risk in the identified areas, such as measles strains, human behavior, population immunity, climatic variables, economic status, and control measures. This information can be used to design effective intervention strategies to reduce and hopefully eradicate the disease in high-risk areas.

In conclusion, PAR%, a method of risk estimate, is an effective way to analyze multiple clusters. It supplements traditional methods of spatiotemporal analysis and also applies to other highly infectious diseases. For measles outbreaks in Shandong Province, temporal and spatial differences prompt us to focus on key areas that are located in the northwest and southwest. Given the high measles mortality rate in infants aged <1 year old and its possible reasons, interrupting measles circulation and maintaining routine coverage over 95% may be the only effective strategy for launching measles elimination. Meanwhile, publicity and education is also very important. We will post the finding of this study to the Shandong CDC's website to let more people know about measles. Therefore, we need to plan local policies for infant measles control in order to meet the target of complete elimination of measles in China, which owes the largest population in the world.
